# Treatment of Cells and Tissues with Chromate Maximizes Mitochondrial 2Fe2S EPR Signals

**DOI:** 10.3390/ijms20051143

**Published:** 2019-03-06

**Authors:** William E. Antholine, Jeannette Vasquez-Vivar, Brendan J. Quirk, Harry T. Whelan, Pui Kei Wu, Jong-In Park, Charles R. Myers

**Affiliations:** 1 Department of Biophysics, Medical College of Wisconsin, Milwaukee, WI 53226, USA; jvvivar@mcw.edu; 2 Departments of Neurology and Pediatrics, Medical College of Wisconsin, Milwaukee, WI 53226, USA; bquirk@mcw.edu (B.J.Q.); hwhelan@mcw.edu (H.T.W.); 3 Department of Biochemistry, Medical College of Wisconsin, Milwaukee, WI 53226, USA; pkwu@mcw.edu (P.K.W.); jipark@mcw.edu (J.-I.P.); 4 Department of Pharmacology and Toxicology, Medical College Wisconsin, Wauwatosa, WI 53226, USA; cmyers@mcw.edu

**Keywords:** electron paramagnetic resonance, EPR, chromate, mitochondria, cells, tissues

## Abstract

In a previous study on chromate toxicity, an increase in the 2Fe2S electron paramagnetic resonance (EPR) signal from mitochondria was found upon addition of chromate to human bronchial epithelial cells and bovine airway tissue ex vivo. This study was undertaken to show that a chromate-induced increase in the 2Fe2S EPR signal is a general phenomenon that can be used as a low-temperature EPR method to determine the maximum concentration of 2Fe2S centers in mitochondria. First, the low-temperature EPR method to determine the concentration of 2Fe2S clusters in cells and tissues is fully developed for other cells and tissues. The EPR signal for the 2Fe2S clusters N1b in Complex I and/or S1 in Complex II and the 2Fe2S cluster in xanthine oxidoreductase in rat liver tissue do not change in intensity because these clusters are already reduced; however, the EPR signals for N2, the terminal cluster in Complex I, and N4, the cluster preceding the terminal cluster, decrease upon adding chromate. More surprising to us, the EPR signals for N3, the cluster preceding the 2Fe2S cluster in Complex I, also decrease upon adding chromate. Moreover, this method is used to obtain the concentration of the 2Fe2S clusters in white blood cells where the 2Fe2S signal is mostly oxidized before treatment with chromate and becomes reduced and EPR detectable after treatment with chromate. The increase of the g = 1.94 2Fe2S EPR signal upon the addition of chromate can thus be used to obtain the relative steady-state concentration of the 2Fe2S clusters and steady-state concentration of Complex I and/or Complex II in mitochondria.

## 1. Introduction

**Figure 1:**
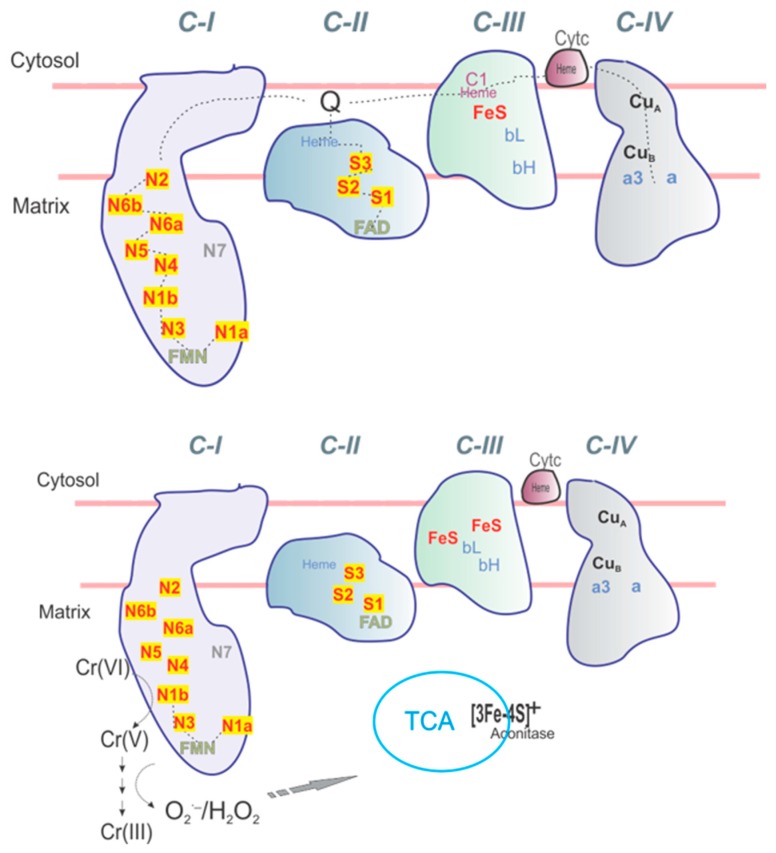
Mitochondrial iron cluster organization and EPR signals. (Upper panel) Mitochondrial Respiratory Complexes (CI–CIV). The hydrophilic domain and iron sulfur clusters of CI: N1a, N1b (2Fe2S) only. N1b is detected at g = 1.94 and 2.02. The clusters N2, N3, N4, N5 are (4Fe4S) with typical signals at g = 2.05 for N2; g = 2.04 and 1.87 for N3; g = 1.89 for N4. Mitochondrial C-II contains S1 (2Fe2S), S2 (4Fe4S) and S3 (3Fe4S) complexes with signals at g = 2.02, 1.93 (S1), g = 2.02 (S3), respectively. (Lower panel) changes in EPR signals upon addition of chromate including contribution of inactive aconitase (3Fe4S)+; g = 2.02. Note: only partial g-values of interest are given for EPR spectra for Fe-S clusters.

Most of the iron sulfur (Fe-S) site signals in mitochondrial Complex I, including the four-iron four-sulfur clusters (4Fe4S) and two-iron two-sulfur clusters (2Fe2S), can be detected using electron paramagnetic resonance (EPR) when these clusters are in the reduced state (Figure 1). Other Fe-S signals, primarily from the three-iron four-sulfur cluster (3Fe4S) in aconitase, are detected by EPR when they are in the oxidized state. An increase in the g = 1.94 EPR signal attributed to 2Fe2S sites in Complex I and/or Complex II in mitochondria upon addition of chromate to human bronchial epithelial cells and bovine airways treated ex vivo is thought to be an important biomarker for Cr(VI) exposure [1]. Recently, it occurred to us that the intensity of this g = 1.94 2Fe2S signal could be more widely used to determine the relative concentration of Complex I and/or Complex II in mitochondria. Even though chromate is toxic at the concentration added to cells and tissues in this study, the idea is to use a saturating amount to determine the concentration of the 2Fe2S sites from the resulting EPR signals. Similarly, while cyanide is toxic, it is frequently used to inhibit Complex IV in mitochondria to study mitochondrial function [2]. Treatment of human bronchial BEAS-2B cells with chromate also results in inhibition of thioredoxin reductase and oxidation of thioredoxins, and mitochondrial thioredoxin-2 is more susceptible than cytosolic thioredoxin-1 [3,4]. If chromate blocks the electron flow in mitochondria after the N1b 2Fe2S cluster in Complex I, its 2Fe2S cluster would be reduced, increasing its g = 1.94 EPR signal, while Fe-S signals for the mitochondrial 2Fe2S clusters downstream of the chromate-induced blockade would be expected to decrease due to the loss of electron flow (i.e., these Fe-S clusters become oxidized and no longer exhibit a paramagnetic signal). As for these downstream Fe-S clusters, greater oxidation of mitochondrial thioredoxin-2 suggests that chromate results in reactions in the mitochondria that increase oxidized states over reduced states [5]. Moreover, chromate disrupts proteins that control thiol redox reactions [5]. 

In this report, chromate toxicity is not emphasized, although the excessive chromate used is expected to be toxic; rather, even though much oxidation is induced by chromate, the emphasis is on the unique ability of chromate to increase the 2Fe2S g = 1.94 EPR signal representing the reduced state of 2Fe2S centers that are upstream of the chromate-induced blockade of mitochondrial electron transport. The signal from these 2Fe2S site(s) is a means to measure the concentration of these 2Fe2S sites, and, in turn, the concentration of Complex I and/or Complex II in mitochondria. This EPR method quantitates Complex I in mitochondria before and after treatment with agents of interest, analogous to other methods that rely on inhibition of mitochondrial electron transport with rotenone, antimycin and so forth. This EPR method, which utilizes the addition of chromate to increase the signals for the upstream 2Fe2S signal and to decrease the signals for downstream 4Fe4S signals, is a relatively easy procedure that does not require the addition of other substrates or the removal of oxygen.

## 2. Results

### 2.1. Increase of the g = 1.94 2Fe2S Signal Upon Addition of Chromate to White Blood Cells

**Figure 2:**
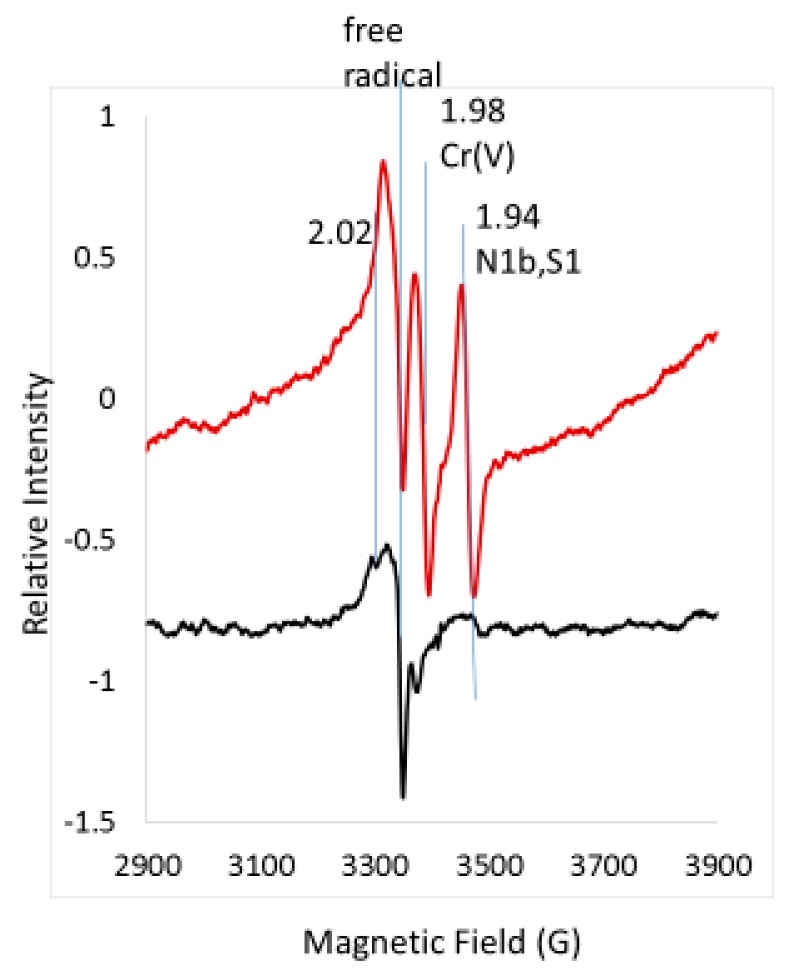
EPR spectra for untreated white blood cells (black) versus those treated with 400 μM chromate (red). Cells were incubated at 37 °C for 3 h. Vertical lines indicate superposition of lines from several sites (g = 2.02), Cr(V) (g = 1.98) and the N1b and S1 2Fe2S centers (g = 1.94–1.93). Spectrometer conditions: microwave freq., 9.387 GHz; temp., 10 K; mod. amp., 5 G; microwave power, 5 mW; sweep time, 83.89 s; time constant, 81.82 ms; nine scans.

EPR spectra were obtained from white blood cells with and without the addition of 400 μM chromate (Figure 2). Of interest for this study is the very substantial increase of the g = 1.94 2Fe2S signal for cells treated with chromate, which reached a signal intensity as large as that detected for chromate-treated human bronchial epithelial cells and bovine airways [1]. The signal at g = 2.02 attributed to the low-field g-value for the 2Fe2S EPR signal also increased but superimposed on it are signals from 3Fe4S sites including the EPR signal for oxidized aconitase, which makes this region of the EPR spectrum difficult to analyze [6,7,8,9,10,11]. The Cr(V) signal at g = 1.98 also was detected in the EPR spectrum in which chromate was added. This signal is attributed to multiple adducts for Cr(V) with proteins and other low molecular weight thiols upon one-electron reduction of chromate to Cr(V) (Figure 1) [12] (and refs therein). The g-value for Cr(V) signals ranges from 1.99 for Cr(V) with sulfur donor atoms to 1.98 for Cr(V) without sulfur donor atoms. An increase in a sharp free radical signal, often assigned to ubisemiquinone [13], was characteristically found in the chromate samples. This is indicative of additional redox reactions occurring due to the addition of chromate. Mitochondria have at least two 2Fe2S sites: one is the N1b site in Complex I with g-values of 2.02, 1.94 and 1.92 and the other is the S1 site in Complex II with g-values of 2.02, 1.93 and 1.91 [11,14]. If the number of cells and the concentration of the 2Fe2S signal are known by comparison to a known concentration of a 2Fe2S signal or the relative steady-state concentration is known by comparison to a standard signal (see the following example for the 2Fe2S signal in liver tissue), the concentration of Complex I is inferred, and an approximation of the electron transport chain concentration can be obtained. For example, the 2Fe2S signal in untreated white blood cells is mostly oxidized compared with the large reduced g = 1.94 signal obtained with chromate treatment.

Chromate is an oxidizing agent that can be reduced by several redox active proteins to Cr(V) and Cr(III) (Figure 1). Evidence of the oxidizing capacity of chromate is the strong signal (g = 6) for high spin ferric heme in white blood cells treated with 400 μM chromate (Figure S1). EPR signals, which are most likely for Cr(V) adducts and Cr(III) adducts, as described in our earlier work [15,16], are also seen, confirming the reduction of Cr(VI) and the formation of thiol and protein adducts of Cr(V) and Cr(III) in these cells (Figure S1).

### 2.2. Chromate Added to Liver Cells

#### 2.2.1. Chromate Does Not Oxidize the 2Fe2S Sites in Liver Tissue

**Figure 3:**
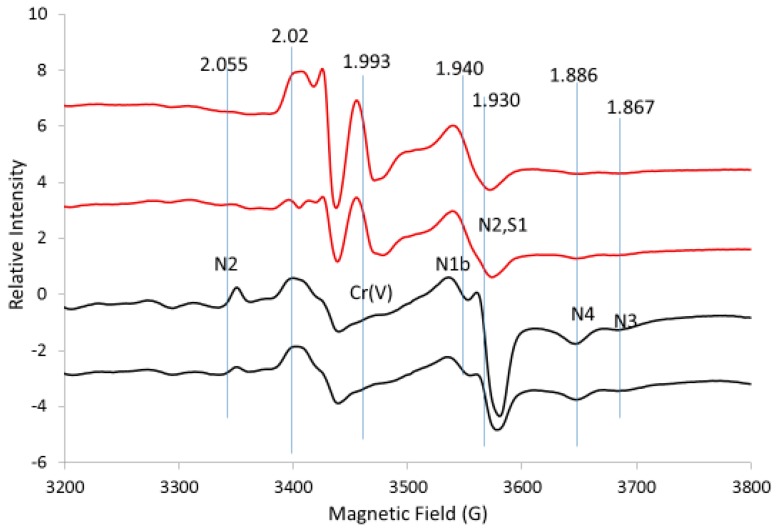
EPR spectra for control liver tissue (black) versus tissue treated with chromate (red). The vertical lines indicate the g-values for EPR lines from Complex I (N2, N1b, N3, N4) and Complex II (S1) discussed in the text. Spectrometer conditions: microwave frequency, 9.632 GHz; temp., 10 K; mod. amp., 5 G; microwave power, 5 mW; sweep time, 83.89 s; time constant, 81.82 ms.

EPR spectra were obtained from rat liver, kidney and heart tissue to determine the intensity of the Fe-S signals (Figure S2). Typical Fe-S signals were found in the heart, which is the most studied tissue for Fe-S signals [8,9,17]. These same Fe-S signals were observed in liver and kidney tissue. Liver tissue was selected for further study because it is easier to perfuse chromate into liver tissue than into heart or kidney tissue. The EPR signals for reduced Fe-S clusters are intense in control liver tissue, indicating that the maximum EPR signals are already attained (Figure 3). As such, little or no increase in the EPR signal was expected upon addition of chromate. The EPR signals we observed in the liver tissue are similar to signals observed by others [11]. The g-values on our figures are the same as g-values obtained by others [4,14]. These g-values are used to assign sites.

EPR spectra (at 10 K) of duplicate control liver tissues without added chromate and liver tissues treated with chromate are shown in Figure 3. More than one 2Fe2S signal at g = 1.94 to g = 1.93 was resolved. Lines at g-values of 2.04, 1.93 and 1.867 were assigned to the 4Fe4S cluster, N3, of Complex I. The 2.04 line is overlapped by a multitude of lines; however, since the baseline is flatter in the spectra of chromate-treated samples, this suggests chromate-induced loss of this line. One of the EPR lines in the cluster of lines at g = 1.93 decreases when chromate is added. The high field line at g = 1.867 is not superimposed by other lines and gives the best indication for the 4Fe4S site, N3. It is concluded that the 4Fe4S signal at g = 1.867 is sensitive to oxidation by chromate and thus is lost when chromate is added.

The EPR lines recorded at 10 K at g-values of 2.02, 1.94 and 1.92 were assigned to N1b, a 2Fe2S site in Complex I. The g = 2.02 line is superimposed by lines from 3Fe4S sites, for which the 3Fe4S signal from aconitase often contributes a major fraction of this signal. Judging from the top of the g = 1.94 line, it remains almost constant with or without chromate and the g = 1.92 line is not resolved from the g = 1.94 line (Figure 3).

This spectral pattern led us to conclude that the 2Fe2S signal intensity for Complex I in liver is not affected by chromate addition, indicating that the signal for N1b in control liver represents the complex in its fully reduced state. EPR lines at g-values of 2.06, 1.93 and 1.90 were assigned to N4 or N5, which are 4Fe4S sites in Complex I [10]. It is noteworthy that the low intensity of the EPR line at g = 1.90 implies that this site is not detected under our experimental conditions.

The EPR lines at g-values of 2.10, 1.94 and 1.89 were assigned to N4(TY)1, a 4Fe4S site in Complex I (14). The line at g = 1.89 is less intense in chromate-treated liver. However, since the line at g = 1.94 is a superposition of many lines, it is not a good indicator of the N4(TY)1 site. It is concluded that the EPR signal for the 4Fe4S site in the reduced state, N4(TY)1, decreases with the addition of chromate.

Lines at g-values of 2.055, 1.92 and 1.92 were assigned to N2, a 4Fe4S site in Complex I. The g = 2.055 line is less intense in chromate-treated liver (Figure 3). The g = 1.92 line is a shoulder on the g = 1.93 and g = 1.94 lines in the liver tissue samples and its intensity is lost upon addition of chromate (Figure 3). The N2 site signal decreases with the addition of chromate as concluded from the loss of the g = 2.055 and g = 1.92 lines. Lines with g-values of 2.02, 1.93 and 1.91 were assigned to S1, a 2Fe2S site in Complex II [10,18,19]. Because these lines are so close to the lines for the 2Fe2S N1b site in Complex I, we are not able to determine how much of the EPR signal is from Complex I versus Complex II. Since many other Fe-S lines are detected from other components of Complex I, it is concluded that N1b, the 2Fe2S signal from Complex I, is a major component of the g = 1.94 signal in liver tissue and does not change upon addition of chromate. Many Fe-S sites have a g-value of about 1.93, so those simulations are not unique. However, the intensity of these lines after addition of chromate decreases and is minimal compared with the intensity of the 2Fe2S lines.

#### 2.2.2. Temperature Dependence for EPR Spectra from Liver Tissue Treated with Chromate

**Figure 4:**
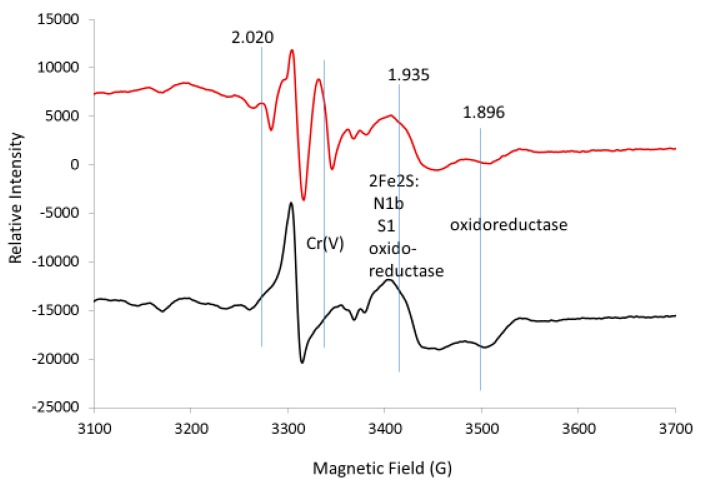
EPR spectra for control liver tissue (black) versus liver treated with chromate (red). The vertical lines indicate the g-values for EPR lines from Complex I (N1b), Complex II (S1) and the 2Fe2S site in xanthine oxidoreductase as discussed in the text. Spectrometer conditions: microwave freq., 9.272 GHz; temp., 110 K; mod. amp., 5 G; microwave power, 20 mW; sweep time, 83.89 s; time constant, 81.92 ms.

**Figure 5:**
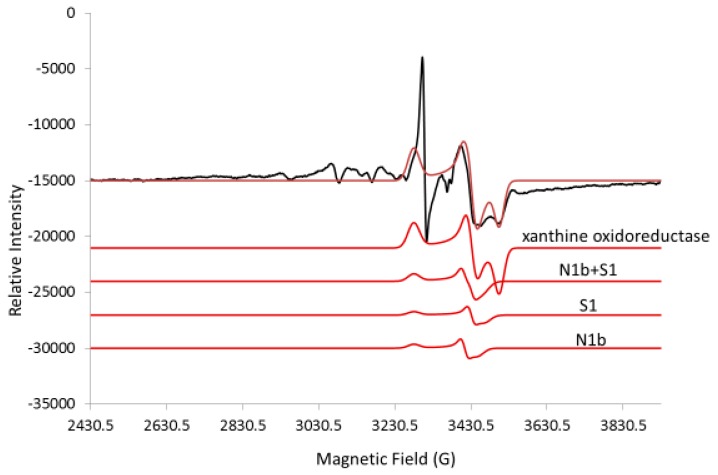
EPR spectrum for control liver tissue (black) at 110 K and simulations (red). Bottom (N1b): g = 2.020, 1.94, 1.92; Hstrain (100, 50, 100). Second from bottom (S1): g = 2.02, 1.93, 1.91; Hstrain (100, 50, 100). Second from top (xanthine oxidoreductase): g = 2.02, 1.93, 1.90; Hstrain (100, 75, 75); lwpp, 0.5. Top (Sum of N1b+S1+xanthine oxidoreductase): microwave freq., 9.272 GHz.

While the previous spectra were collected at 10 K, only the EPR spectra for the 2Fe2S signals in the g = 1.94–1.93 region were detected at 110 K (Figure 4); this is because the signals for 4Fe4S and 3Fe4S are broadened at 110 K and do not interfere. The g = 1.94 line has shoulders that suggest a superposition of at least two lines. Partial resolution of a line with the proper shape at g = 1.92 was assigned to the reduced N1b 2Fe2S site of Complex I, which confirms that part of the g = 1.94 line is due to this 2Fe2S site in Complex I. There is also a well-resolved line at g = 1.90 in the spectra obtained at 110 K; this is characteristic of a 2Fe2S signal from xanthine oxidoreductase with reported g-values of 2.02, 1.93 and 1.90 in samples prepared from liver tissue [20,21]. The intensity of the g = 2.02 line is diminished in the 110 K spectra (Figure 4) due to the loss of 3Fe4S signals at higher temperatures. At 110 K, the free radical signal increased as this signal was less saturated and the signal at g = 1.99 in chromate-treated liver is due to Cr(V) adducts. From these 110 K spectra, it is concluded that a third 2Fe2S signal tentatively assigned to xanthine oxidoreductase contributes at g = 2.02 and g = 1.93, further complicating the spectra in these regions. The lines in the g = 1.94–1.91 region were simulated by adding simulated spectra for the 2Fe2S sites in N1b, S1 and xanthine oxidoreductase; they nicely fit the experimental spectrum (Figure 5). The 2Fe2S signals from N1b, S1 and xanthine oxidoreductase after addition of chromate remained intense at 110 K (Figure 5), whereas the 2Fe2S signal from xanthine oxidoreductase after treatment with chromate was weak at 10 K under our conditions (Figure 3).

**Figure 6:**
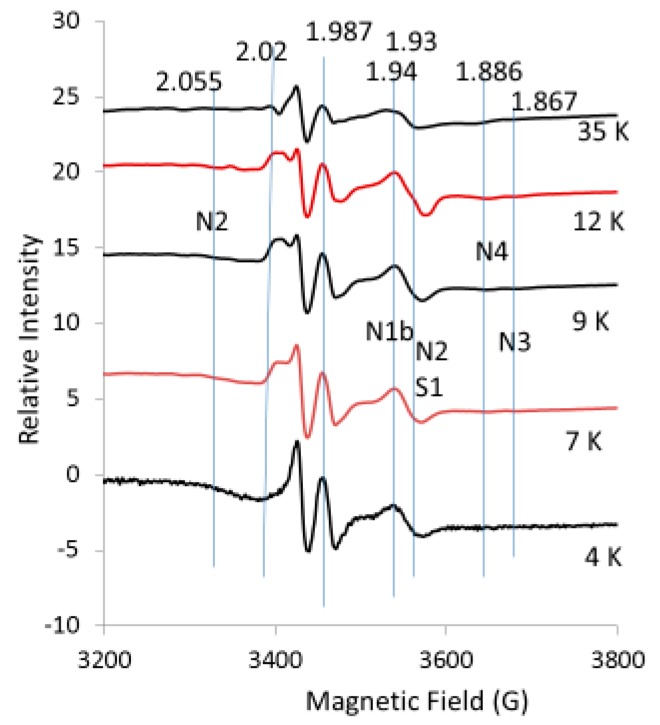
EPR spectra for control liver tissue at 4 K, 7 K, 9 K, 12 K and 35 K. The vertical lines indicate the g-values for EPR lines from Complex I (N2, N1b, N4) and Complex II (S1) discussed in the text. Spectrometer conditions: microwave freq., 9.632 GHz; mod. amp., 5 G; microwave power, 5 mW.

Overall, it is concluded that all the 4Fe4S and 3Fe4S signals are less intense due to the broadening of these signals at 110 K (compared to 10 K) in Complex I and due to the treatment with chromate. Only the 2Fe2S signals remained constant in Complex I and/or Complex II and in xanthine oxidoreductase in the liver tissue. It is known from previous work that the activity for both Complex I and Complex II is lost upon addition of chromate to human bronchial epithelial cells [1]. The line shape of the g = 1.94 signal changed with temperature (Figure 6) and the g = 1.94 line is even more symmetrical at 4 K and 7 K. At 12 K, there is an inflection at the center of the S-shaped line. At 35 K and 110 K, there is a shoulder on the high field of the line in addition to the inflection at the center of the g = 1.94 line (Figure 4 and Figure 6). The saturation behavior of the g = 1.94 line from N1b differs substantially when a nearby 4Fe4S site is oxidized or reduced, further complicating the temperature-dependence analysis [7,22]. Since no 4Fe4S and 3Fe4S signals exist at the higher temperatures, the signals at 110 K are consistent with at least three superimposed lines at g = 1.94, assigned to the 2Fe2S sites in N1b in Complex I and S1 in Complex II and the 2Fe2S site in xanthine oxidoreductase, which is more evident at the higher temperatures. 

#### 2.2.3. Increase of low-field EPR Lines Due to Oxidation by Chromate

**Figure 7:**
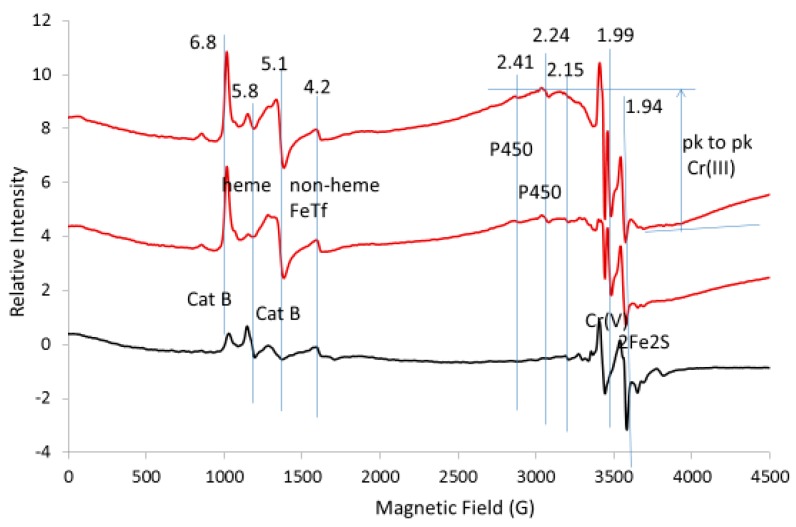
EPR spectra for control liver tissue (black) versus tissue treated with chromate (red). The vertical lines indicate the g-values for EPR lines from Complex I (primarily N1b) and Complex II (S1) as discussed in the text. Spectrometer conditions: microwave freq., 9.632 GHz; temp., 10 K, mod. amp., 5 G; microwave power, 20 mW; sweep time, 83.89 s; time constant, 81.82 ms.

An increase in the low-field lines in the expanded EPR spectrum at 10 K is consistent with the overall oxidizing properties of chromate (Figure 7). High spin catalase lines at g = 6.8 and g = 5.1 increased several-fold with chromate treatment [8,11]. Lines at g = 2.41 and g = 2.25 were assigned to heme in cytochrome P450; these lines also increased several-fold with chromate. The high-field S-shaped line at g = 1.81 was tentatively assigned to a two-iron center, possibly the two-iron center in myo-inositol oxygenase [23]; its g-values at 1.95 and 1.81 disappeared with chromate, presumably due to its oxidation to a non-EPR active state. EPR lines for Cr(III) adducts (as marked) and Cr(V) adducts (g = 1.99) appear in the chromate-treated tissue (Figure 7). The sharp line at g = 1.99 is thought to be a superposition of many Cr(V) adducts and the broad line indicated by the horizontal lines at the maximum and minimum of the line is thought to be a superposition of many Cr(III) adducts with small zero field splitting values. No intense lines were observed at low fields indicating Cr(III) adducts with a large zero field splitting. Changes in the peak-to-peak height for these signals confirm that chromate is being reduced to Cr(V) and Cr(III) adducts and the other signals indicate that many sites throughout the cell are being oxidized.

#### 2.2.4. Determining the Concentration of the Mitochondrial 2Fe2S Cluster in Liver Tissue

**Figure 8:**
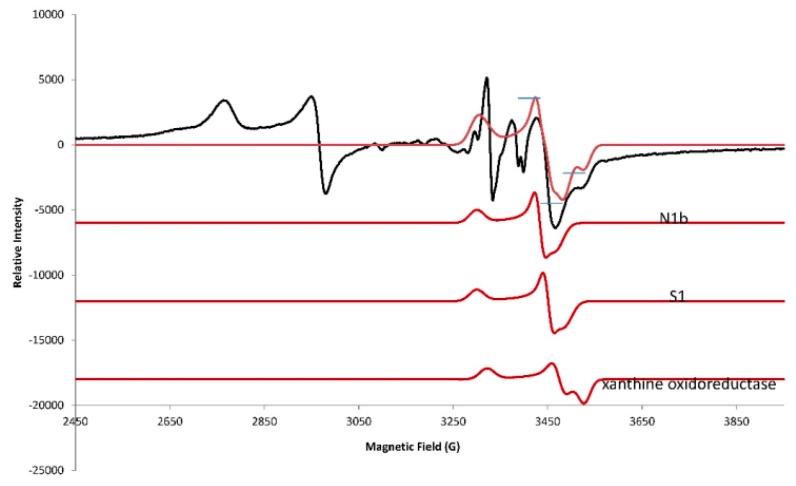
Simulation of 2Fe2S for N1B, S1, oxidoreductase. EPR spectrum for liver tissue sample (black) at 110 K and simulations for 2Fe2S clusters (red). Bottom (xanthine oxidoreductase): g = 2.02, 1.93, 1.90; Hstrain (100, 75, 75). Second from bottom (S1): g = 2.02, 1.93, 1.91; Hstrain (100, 50, 100); Second from top (N1b): g = 2.020, 1.94, 1.92; Hstrain (100, 50, 100); lwpp, 0.5. Top (sum of N1b+S1+xanthine oxidoreductase): microwave freq., 9.272 GHz. Note: The peak height for the line at g = 1.92 is a good marker for the 2Fe2S signal in xanthine oxidoreductase and the peak-to-peak height in the g = 1.94–1.93 region is a good marker for the sum of the signals from the three 2Fe2S clusters.

The EPR spectrum in the g = 1.94 region for liver tissue was simulated by adding simulated spectra for the 2Fe2S signal from N1b, S1 and xanthine oxidoreductase (Figure 8). The peak-to-peak height for the g = 1.94–1.93 lines is fit and the baseline-to-peak height for the g = 1.90 line from xanthine oxidoreductase is fit. The base lines differ, probably because of contributions from other EPR detectable sites with a g-value of 1.94–1.93 and broad lines were not considered. Overall, the fit allows for a rough assignment of the major components but inclusion of simulations for these minor components with respect to signal height should improve the fit. Here, the rough fit is used to show how to obtain the concentration. The simulated fit was double integrated (Figure S3) and compared with the double integral from a standard EPR signal (1.0 mM CuEDTA (not shown), for example). It is suggested that a weaker signal of, for example, purified Complex I would be a better standard.

Comparison of the ratio for the sum of the double integral for N1B, S1 and xanthine oxidoreductase with the double integral from the standard gives 9 μM total and thus 3 μM each for the 2Fe2S clusters N1B, S1 and oxidoreductase (Figure 8). The relative comparison between the 2Fe2S signals in Figure 5 and Figure 8 is consistent with a concentration of 3 μM each for the 2Fe2S clusters in N1b and S1 and 5.4 μM for the 2Fe2S cluster in xanthine oxidoreductase using the assumptions outlined (Figure S3). Once the concentrations of these signals are known, all other signals can be compared through simulation and double integration of the individual clusters.

### 2.3. Use of the 2Fe2S Signal Obtained from Addition of Chromate to Melanoma Cells as An Internal Control

**Figure 9:**
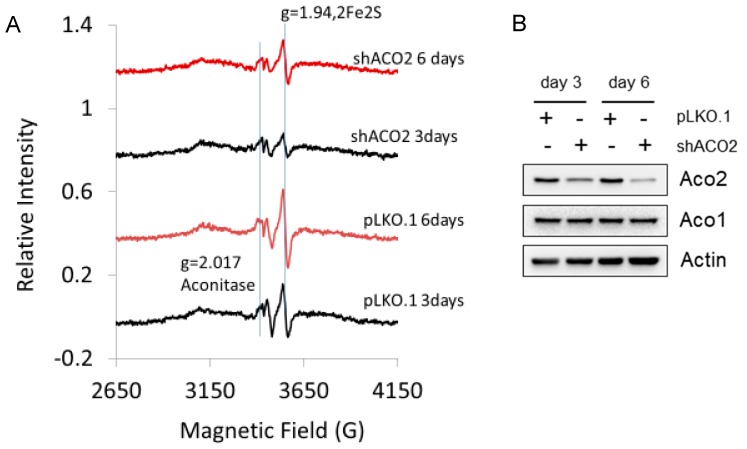
(**A**) A375 melanoma cells depleted of mitochondrial aconitase (shACO2) for three days (second from top spectrum, black) and six days (top spectrum, red). All cells were treated with 10 µM chromate for 30 min before freezing. pLKO.1 is the control for shACO2. Spectrometer conditions: microwave freq., 9.632 GHz; mod. amp., 5 G; temp., 8.8 K, microwave power, 16 dB; (**B**) Western blot analysis of mitochondrial aconitase (ACO2) and cytosolic aconitase (ACO1) levels in A375 cells infected with lentiviral shACO2 or the control pLKO.1. Actin was the control for equal amounts of protein loading.

In order to resolve the difficulty of 3Fe4S sites attributed to aconitase, we knocked down mitochondrial aconitase using an shRNA system. Most of the Fe-S signals in the EPR spectrum for untreated melanoma cells are absent presumably because the Fe-S sites are oxidized. This exemplifies that little EPR information is gained when these Fe-S centers are mostly oxidized. Nevertheless, the addition of chromate increased the 2Fe2S signal at g = 1.94, consistent with our data for white blood cells (Figure 2) and our prior data for bronchial epithelial cells and airway tissues [1]. Before the addition of chromate to melanoma cells, there was a weak 2Fe2S signal and a 3Fe4S signal assigned to oxidized aconitase (spectra not shown). Upon addition of chromate, the 2Fe2S signal increased and the 3Fe4S from aconitase decreased presumably due to further oxidation to EPR silent states (not shown). The spectrum for the 2Fe2S site could be simulated and g = 2.02 could be subtracted, leaving only the 3Fe4S signal, which includes the signal for aconitase, especially if the aconitase signal is weak. The peak-to-peak height of the 2Fe2S signal at g = 1.94 (Figure 9) was used to determine the 2Fe2S concentration by taking the relative peak heights for the g = 1.94 signal. This signal is proportional to the concentration of Complex I and/or Complex II, which is proportional to the concentration of mitochondria in the cells. On day 3, there were 4.8 × 10^5^ pLkO.1 cells/mL and on day 6, there were 9.1 × 10^5^ cells/mL. On day 3, there were 2.8 × 10^5^ shACO2 cells/ml and on day 6, there were 4.7 × 10^5^ shACO2 cells/mL. The intensity of the g = 1.94 line for the 2Fe2S clusters nicely correlates with the number of cells (Figure 9). This can be valuable information in that the Fe-S sites are oxidized in the absence of chromate and in the presence of chromate the mitochondria are clearly accounted for, not absent. Nevertheless, aconitase knockdown revealed only a mild difference in the presence of chromate where the change was expected at day 6. 

## 3. Discussion

Accurately quantifying Complex I and/or Complex II content in mitochondria can provide valuable information. The 2Fe2S signal can be used as a normalizing factor for the variability in mitochondrial content in biological samples [24]. Complex I, which is the first complex in the electron transport chain, is responsible for approximately one-third of all mitochondrial respiratory chain deficiencies. Complex I deficiency is a progressive neurodegenerative disorder and causes various clinical symptoms in tissues such as brain, heart, liver and skeletal muscle. Our study included liver tissue because it has strong EPR signals from Fe-S clusters. Major forms of Complex I deficiency include myopathy (muscle disease), mitochondrial encephalomyopathy (brain and muscle disease) and fatal infantile multisystem disorder. Leher’s hereditary optic neuropathy, MELAS (mitochondrial encephalopathy, lactic acidosis, stroke-like episodes), MERRF (myoclonic epilepsy with ragged red fibers) and Leigh Syndrome, also are associated with Complex I deficiency. We focus on the signal from the 2Fe2S clusters of Complex I and/or Complex II. Although both Complex I deficiency and Complex II deficiency are due to autosomal recessive causes and have no cure, it seems reasonable that having another method to determine the 2Fe2S clusters in Complex I and Complex II would be a useful tool that could be translated to clinical use.

In this paper, an EPR method was developed to demonstrate how the addition of chromate increases the 2Fe2S signals from Complex I and/or Complex II. The intensity of this EPR signal can be used to determine indirectly the concentration of Complex I and/or Complex II, which relates to the concentration of mitochondria in the cells. It is noted that when using this method, the researcher needs to repeat the experiments to get valid statistical results. The correlation of peak-to-peak signal height for the 2Fe2S signal versus the number of cells per milliliter in Figure 9 attests to the accuracy of the EPR method. It is our experience that the signals from the uncalibrated tubes and the samples run on different days can vary by +/−15% but often the percentage is much less. We can be more confident in changes in peak height if some peaks increase and others decrease. This study shows only that addition of chromate increases the 2Fe2S signal in three types of cells and that there is no loss of signal in liver tissue. Although the 2Fe2S signal gives the concentration of Complex I and/or Complex II, the stoichiometry of Complexes I–IV is not simply 1:1. The results of these experiments suggest a method to quantitate the 2Fe2S signal and can be improved upon by individual investigators. We simply used CuEDTA as a standard to measure the concentration of the spins but more appropriate standards could be used; for example, Complex I could be purified and completely reduced. The simulations in Figure 5 and Figure 8 illustrate the method using the most dominant signals and could be improved by adding other Fe-S signals that contribute less substantially.

Since xanthine oxidoreductase has a well-resolved line with a low-field g-value of 1.90 at 110 K, the line can be fit and the concentration of the 2Fe2S site for xanthine oxidoreductase can be determined. The contribution to the signal of the 2Fe2S site at 10 K from xanthine oxidoreductase upon addition of chromate, however, is lost and therefore not included in the simulations at 10 K. This indicates that chromate does not oxidize mitochondrial 2Fe2S sites and, in fact, captures electrons in the reduced 2Fe2S sites and increases the EPR signal intensity when much of the 2Fe2S cluster in cells or tissues is oxidized (for example, Figure 2). At 10 K, where the 2Fe2S signal from xanthine oxidoreductase contribution to the signal is negligible, the intensity of the g = 1.94 signal can be used as a measure of the concentration of Complex I and/or Complex II, where the top of the S-shape is more indicative of the concentration of Complex I. Reduction of chromate to Cr(V) and/or Cr(III) in the mitochondria likely results in thiol adducts that may occur at or near the 2Fe2S site of Complex I. Consistent with a chromate-induced electron transfer blocked at the 2Fe2S site or after the 2Fe2S site but before the closest 4Fe4S site, the 4Fe4S site is oxidized for all or most of the sample, as determined by a loss of the EPR signal. As judged by the oxidized state of Fe-S clusters after the 2Fe2S site, it appears that there is little reverse electron transfer, where electrons are transferred backwards from Complex II [25]. In addition to blocking electron transport resulting in isolation of the 2Fe2S site in a reduced state, it is possible that the 2Fe2S clusters are not solvent accessible and, thus, are protected from direct oxidation by oxidizing agents. Inconsistent with blockage of electrons at or about the 2Fe2S site is that the signal for N3, a 4Fe4S site between flavin and the 2Fe2S site is also decreased with chromate. Another possibility is that electrons leak from the N3 cluster when transport through Complex I is inhibited. Since at least three signals are within g = 1.94–1.93, the components from N1b of Complex I, a second component from the S1 site of Complex II and a weak contribution at 10 K but a stronger contribution at 110 K from the 2Fe2S signal of xanthine oxidoreductase, are accounted for.

Here, we show EPR-based methodology for the quantification of mitochondrial 2Fe2S clusters and, by inference, mitochondria. The EPR signals from the 2Fe2S clusters in liver tissue are already close to maximum; the signals increase with the addition of chromate, when the 2Fe2S cluster is not fully reduced. Thus, maximizing the 2Fe2S EPR signal by adding chromate to an 2Fe2S EPR signal not at maximum will quantitate the concentration of mitochondria with the reservation that the concentrations of Complexes I–IV may not be equal. The concentration of mitochondria may prove important in the normalization of EPR data obtained at low temperatures and is critical to a better understanding of the significance of variations in EPR signal intensity.

## 4. Materials and Methods

### 4.1. Sodium Chromate

Sodium chromate was of the highest purity available from Aldrich Chemical (Milwaukee, WI, USA).

### 4.2. Liver Tissue

Liver tissue was obtained from C57BL6 mice that were kept on a 12-h light-dark cycle in a temperature-controlled room and received a standard rodent maintenance diet and water ad libitum. The mice were obtained from The Jackson Laboratory (Bar Harbor, ME) and housed in the Biomedical Resource Center animal facility of the Medical College of Wisconsin (Milwaukee, WI, USA). The animal care and all experimental protocols were approved by the Institutional Animal Care and Use Committee of the Medical College of Wisconsin (protocol AUA397 approved August 2016) and conformed to the *Guide for the Care and Use of Laboratory Animals* [26]. Immediately after cervical dislocation euthanasia, livers were first perfused with cold phosphate buffered saline (PBS) and then by chromate solution. After excision, tissue incubations with chromate solutions were completed at room temperature in a slow-rotating surface.

### 4.3. White Blood Cells

Whole blood was obtained from the BloodCenter of Wisconsin (Milwaukee, WI, USA). One unit of blood was diluted 1:1 with PBS. The diluted blood was layered onto a Ficoll gradient and spun at 1200 rpm for 30 min. The peripheral blood mononuclear cell layer was collected by pipette, washed with PBS, reconcentrated by centrifugation and resuspended in RPMI medium at a concentration of 1 × 10^6^ cells/mL. The white blood cells were incubated with either saline (control) or chromate (final concentration of 400 μM) for 3 h at 37 °C before being loaded into EPR tubes, frozen in liquid nitrogen and stored in either liquid nitrogen or at −80 °C in a Revco freezer.

### 4.4. Melanoma Cells, Virus Infection, Western Blotting

The human melanoma cell line A375 was obtained from ATCC (Manassas, VA, USA). Cells were cultured in Dulbecco’s minimal essential medium (Invitrogen, Carlsbad, CA, USA) supplemented with 10% fetal bovine serum, 100 U/mL of penicillin and 100 µg/mL of streptomycin. A375 cells were infected with the lentiviral pLKO.1-shACO2 (TRCN0000056561, Dharmacon, Lafayette, CO, USA) to suppress aconitase-2 or the control pLKO.1 for 3 or 6 days prior to EPR. Lentivirus production and infection procedures were previously described [27,28,29]. Cells were treated with 10 µM chromate (Sigma, St. Louis, MO, USA) in HBSS (Invitrogen) in a CO_2_ incubator at 37 °C for 30 min and were harvested using cell scrapers. Cells were then collected by centrifugation, washed in ice-cold 1× PBS, resuspended in 0.3 mL 1× PBS, loaded into EPR tubes and snap-frozen. Cells were also harvested, counted and lysed in 62.5 mM Tris (pH 6.8) –2% SDS mixed with protease and phosphatase inhibitor cocktail (Sigma). Protein levels of ACO2, ACO1 and actin were determined by Western blot analysis as previously described [27,28,29]. 

### 4.5. EPR Equipment

EPR samples of cells and tissues were frozen in 4 mm outside diameter quartz tubes and kept either in liquid nitrogen or at −80 °C in a Revco freezer. For this study, the EPR tubes were not calibrated. Future, more precise studies should use calibrated tubes; however, even using uncalibrated tubes, the effect of chromate on the 2Fe2S signal clearly is substantial. Also, it is reported that the reduction of N1b, 2Fe2S, is sluggish [10] and may not be fully reduced but N1b appeared to be reduced in our studies. Investigators can run additional experiments to obtain the best conditions for a full reduction of 2Fe2S clusters. EPR spectra were obtained at liquid helium temperature (4 K to 35 K) using a Bruker E600 EleXsys spectrometer with an Oxford Instruments ESR-900 helium flow cryostat and either a Bruker DM0101 cavity or a Bruker ER4112SQG cavity. EPR spectra at 110 K were obtained on a Bruker EMX spectrometer. We ran the samples at three microwave powers: 10 dB, 16 dB and 30 dB. The best results considering signal-to-noise ratio at 10 K were at 16 dB, where the 2Fe2S signal is slightly saturated at 10 K but not at 110 K.

Spectrometer conditions are given in the figure legends. A background signal from frozen water was subtracted. Spectra were simulated with EasySpin [30]. Lines were broadened with HStrain but technically the mechanism that should be use for broadening is g-strain.

## Supplementary Materials

Supplementary materials can be found at https://www.mdpi.com/1422-0067/20/5/1143/s1.

## Abbreviations

EPR	Electron paramagnetic resonance
Fe-S	Iron-sulfur cluster
PBS	Phosphate buffered saline
2Fe2S	Two-iron two-sulfur cluster
3Fe4S	Three-iron four-sulfur cluster
4Fe4S	Four-iron four-sulfur cluster
