# Supplementary material for: Treatment of Cells and Tissues with Chromate Maximizes Mitochondrial 2Fe2S EPR Signals
Source: Int J Mol Sci. 2019 Mar 6;20(5):1143. doi: 10.3390/ijms20051143 (PMC6429069; doi:10.3390/ijms20051143)
Supplement: Supplementary file 1 [file ijms-20-01143-s001.pdf]

International Journal of *Molecular Sciences*

MDPI

Article – Supplementary Materials

Treatment of Cells and Tissues with Chromate Maximizes Mitochondrial 2Fe2S EPR Signals

William E. Antholine [1,*], Jeannette Vasquez-Vivar [1], Brendan J. Quirk [2], Harry T. Whelan [2], Pui Kei Wu [3], Jong-In Park [3], and Charles R. Myers [4]

[1] Department of Biophysics, Medical College of Wisconsin, Milwaukee, WI 53226, USA; jvvivar@mcw.edu
[2] Departments of Neurology and Pediatrics, Medical College of Wisconsin, Milwaukee, WI 53226, USA; bquirk@mcw.edu; hwhelan@mcw.edu
[3] Department of Biochemistry, Medical College of Wisconsin, Milwaukee, WI 53226, USA; jipark@mcw.edu; pkwu@mcw.edu
[4] Department of Pharmacology and Toxicology, Medical College Wisconsin, WI 53226, USA; cmyers@mcw.edu
[*] Correspondence: wantholi@mcw.edu; Tel.: +1-414-955-4032

Supplementary Figures

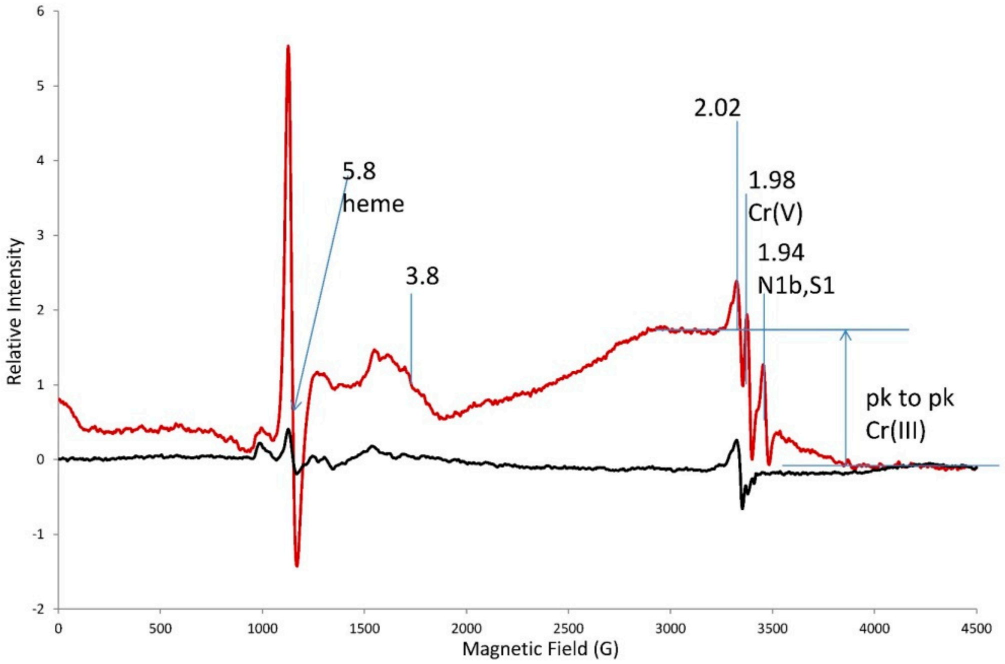

Figure S1. EPR spectra for white cells (black) plus 400 μM chromate (red). Cells incubated at 37 °C for 3 h. Vertical lines indicate g-values for heme (5.8), a line not assigned (3.8), lines from a superposition of several sites (2.02), Cr(V) (1.98), and N1b and S1 (1.94–1.93). The arrow indicates the peak-to-peak height. This signal is tentatively assigned to superposition of Cr(III) complexes. (Note: The line shape suggests absorption or passage conditions.) Spectrometer conditions: microwave freq., 9.387 GHz; temp., 10 K, mod. amp., 5 G; microwave power, 5 mW; sweep time, 83.89 s; time constant, 81.82 ms; nine scans.

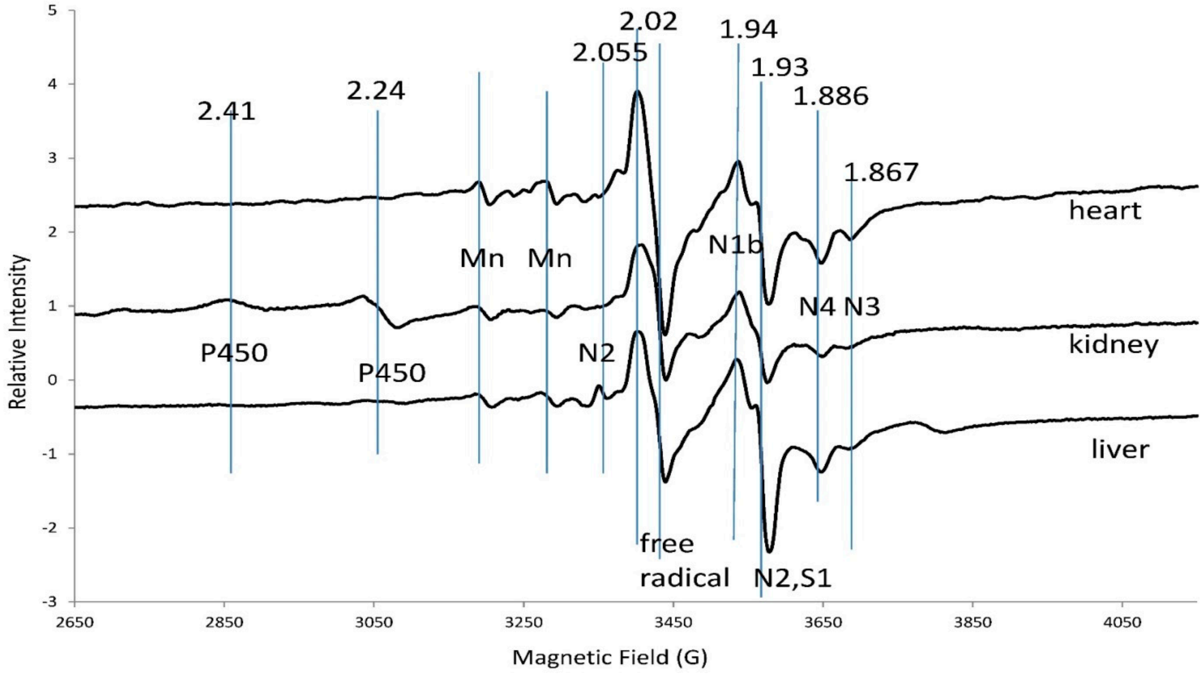

Figure S2. EPR spectra for liver tissue (bottom), kidney tissue (middle), and heart tissue (top). The vertical lines indicate the g-values for EPR lines from Complex I (N2, N1b, N3, N4) and Complex II (S1).

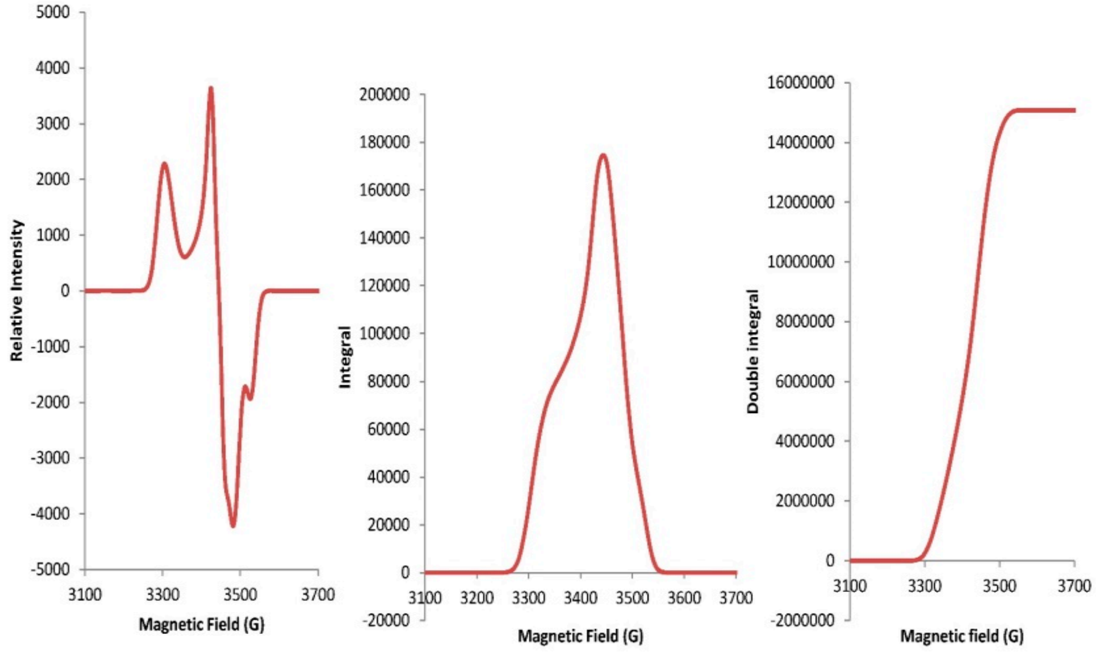

Figure S3. Left: Simulation (EasySpin) for the sum of signals for N1b, S1, and 2Fe2S from xanthine oxidoreductase. Middle: Integral using Sumspc. Right: Double integral using Sumspc. Comparison with a signal from CuEDTA gives a concentration of 3 μM each for N1b, S1, and the 2Fe2S signal for xanthine oxidoreductase.

© 2019 by the authors. Licensee MDPI, Basel, Switzerland. This article is an open access article distributed under the terms and conditions of the Creative Commons Attribution (CC BY) license (http://creativecommons.org/licenses/by/4.0/).
